# A Technology-Enhanced Medical Nutrition Therapy and Diabetes Self-Management Education for Adults With Disability and Type 2 Diabetes: Protocol for a Pilot and Feasibility Randomized Controlled Trial

**DOI:** 10.2196/71495

**Published:** 2025-09-26

**Authors:** Anita Aboagye, Jessica Peckham, Kristine Ria Hearld, Shireen Abdullah, Mohanraj Thirumalai

**Affiliations:** 1 Department of Community Health and Human Services School of Education and Human Sciences University of Alabama at Birmingham Birmingham, AL United States; 2 Kamin Consulting Inc Texas, TX United States; 3 Department of Health Services Administration School of Health Professions University of Alabama at Birmingham Birmingham United States; 4 Division of General Internal Medicine and Population Sciences Heersink School of Medicine University of Alabama at Birmingham Birmingham United States

**Keywords:** diabetes mellitus, telehealth, persons with disabilities, diabetes self-management education, medical nutrition therapy

## Abstract

**Background:**

Diabetes mellitus (DM) is a serious chronic disorder that affects many individuals globally, particularly persons with disabilities, and has long-term adverse effects on the health of individuals and society. Effective self-management education is therefore required. Diabetes management focused on medical nutrition therapy (MNT) and diabetes self-management education (DSME) combined with telehealth technology has the potential to increase the active performance of diabetes management behaviors among persons with disabilities and improve their overall quality of life and quality of self-care.

**Objective:**

This study aims to evaluate the impact of different levels of technology on the delivery of MNT and DSME among persons with disabilities.

**Methods:**

The study is a single-blinded, 3-arm, randomized controlled trial among adults living with both type 2 diabetes and a permanent physical disability. Web-based recruitment is done through partner organizations. The target sample size is 90 participants randomized into 3 arms: a high-technology, a low-technology, and an attention control arm. The high-technology arm receives diabetes-related materials weekly through mediums such as email, a telehealth platform, and text; the low-technology arm receives only 1 weekly email with diabetes-related material; and the attention control arm has no technology support. The intervention is provided by a certified diabetes care and education specialist. Using multivariate linear mixed models, the study examines the relationships between the level of technology intervention and DM self-management behaviors, self-efficacy, and reductions in glycated hemoglobin (HbA_1c_). The primary outcome is the proportion of participants in each group with improved self-management behaviors, as measured by several validated questionnaires. The secondary outcome is a better HbA_1c_ reduction. Outcomes are measured at baseline and at 6 months. Questionnaires and HbA_1c_ measures will be used to measure outcomes.

**Results:**

Data collection began in June 2024 with a total of 90 recruited participants. The intervention was delivered. Make-up classes were delivered to participants in any of the 3 cohorts between November 2024 and December 2024. The final 3-month follow-up classes were held for each cohort 3 months after the first class. Data analysis is anticipated to be completed in fall 2025.

**Conclusions:**

Effective self-management in DM is important to reduce complications. Using technology to deliver MNT and DSME could serve as an effective and convenient strategy for providing these interventions. However, intervention studies are required to determine the most effective level of technology for delivering MNT and DSME intervention to this target group. The YumABLE study is expected to provide new, meaningful, and detailed information about the effectiveness of technology and telehealth platforms for effectively delivering an MNT and DSME program for people living with a permanent physical disability and type 2 diabetes. The results will further improve web- and technology-based diabetes self-management interventions for people with disabilities.

**Trial Registration:**

ClinicalTrials.gov NCT06049225; https://clinicaltrials.gov/study/NCT06049225

**International Registered Report Identifier (IRRID):**

DERR1-10.2196/71495

## Introduction

Diabetes mellitus is a serious chronic disorder that has a significant adverse effect on the overall health of families, individuals, and societies worldwide [1]. According to the International Diabetes Federation, the global prevalence of diabetes among adults aged 20-79 years was approximately 537 million in 2021 [2], representing 10.5% of the global adult population [2,3]. This number is projected to rise to 783.2 million (12.2%) in 2045 [3].

In the United States, DM is one of the most prevalent metabolic conditions, which impacts about 38.4 million of the overall US population (as of 2021), representing 11.6% of the total population. Of this number, 8.7 million are unaware and undiagnosed [4]. Moreover, 97.6 million US adults aged 18 years and older have prediabetes, which usually leads to diabetes [4]. In 2022, the total estimated costs, both direct and indirect, associated with diagnosed diabetes in the United States amounted to US $413 billion [4]. Type 2 diabetes (T2D) can lead to cardiovascular disease, blindness, peripheral neuropathy, and reduced quality of life [5] and is linked with significant morbidity rates [6]. DM is a health concern among all populations; however, persons with disabilities are more likely to be affected by diabetes compared to those without such disabilities [7-10].

Diabetes management primarily relies on individuals and their families, making self-management fundamental in diabetes care [11]. T2D management involves a range of actions and choices such as meal planning, scheduled physical activity, monitoring blood glucose levels, taking diabetes medications, and effectively managing episodes of illness and low and high blood sugar levels [11,12]. These behaviors are meant to regulate blood sugar levels, reduce the risk of complications, and improve the overall health of individuals with diabetes [13].

When individuals with diabetes actively participate in their own care, they can significantly impact both the progression and development of their disease [12]. However, a significant barrier to achieving proper glycemic control appears to be the patient’s inability to maintain adequate self-care, such as poor adherence to prescribed medications and lifestyle adjustments [14]. Thus, educating individuals to self-manage their diabetes yields better health results [15,16].

The literature highlights the potential for diabetes to cause disability; however, the reverse is also possible [17]. Living with disability is associated with an increased risk of DM. A study found that mild and incident disability was associated with 28% and 40% increased diabetes risk, respectively [18]. Diabetes is a significant health concern across all populations; however, persons with disabilities are more likely to be affected. The Centers for Disease Control and Prevention points out that in 2020, approximately 16.2% of persons with disabilities in the United States had received a diagnosis of diabetes, in contrast to 7.5% of those without disabilities [9]. Persons with disabilities are at a greater likelihood of experiencing the major risk factors associated with diabetes [19] due to challenges in maintaining a healthy diet and lifestyle as well as accessing preventive care [20,21].

Physical disability broadly includes conditions affecting mobility like spina bifida and muscular dystrophy [22]. People who are blind, deaf, missing limbs, partially or completely paralyzed, or have other permanent or chronic physical limitations are considered to have a physical disability [23]. Accessibility and safety are the primary concerns with physical disability, with common issues including accessing public areas like city streets, sidewalks, ramps, and public buildings [23]. Some of these disabilities require mobility devices like prostheses, orthoses, canes, wheelchairs, crutches, or walkers [23].

While effective diabetes management is significant in enhancing overall health, persons with disabilities often face barriers that hinder their diabetes self-management [24] and usually exhibit poorer self-management behaviors [24-29]. For instance, self-management behavior was found to be poor among individuals with diabetes and physical limitations [28]; they are less likely to engage in moderate to vigorous physical activity or adhere to their medication compared to those without physical limitations [28]. Individuals with mobility difficulties may encounter challenges attending health care provider appointments related to their diabetes [28]. A study discovered that about 11% of adults with mobility disability had not visited a health professional for their diabetes within the past year [25]. Persons living with physical disabilities in general encounter several difficulties in maintaining a healthy diet [27]. A study found that factors including knowledge, fatigue, health condition, and function impairments influenced the dietary behavior of wheelchair users [27]. Similarly, Hall et al [30] discovered that the expense of healthy foods, a lack of motivation, shopping challenges, and time constraints for shopping or food preparation are significant barriers to maintaining a healthy diet among women with physical disabilities. Nonetheless, the authors noted that about 90% of participants were interested in improving their dietary habits [30]. Another study examining the nutritional status of individuals with spinal cord injury revealed significant deficiencies in essential nutrients, notably fiber, calcium, fruits, and dairy products [31].

Moreover, the participation of persons with disabilities in self-management interventions is limited [24], and their specific self-management needs and capabilities are unrecognized by health care professionals [24,25,29]. It was discovered that 47% of adults with disabilities have not received education on managing diabetes [25]. Age, gender, lack of accessibility, and low socioeconomic status have been found to impact diabetes self-care behaviors among persons with disabilities [24]. In addition, for individuals who are visually impaired, factors such as poor health, concerns about disease advancement, feelings of grief, and experiences of loss inhibit their engagement in diabetes self-care behaviors [29]. Taken together, these barriers can make self-management more challenging particularly for individuals with a physical disability and T2D.

A systematic review of factors influencing the adoption of diabetes management technologies identified monthly income, gender, age, educational level, and diabetes-related characteristics as key determinants of their use; however, of all 28 studies included in the review, disability status was not considered as a factor that could influence technology use in diabetes management [32]. There is limited technology-based diabetes self-management tailored specifically for persons with disabilities [33].

Recently, digital health technologies have progressively been integrated into diabetes education, functioning as either an adjunct to or a substitute for traditional in-person methods [34]. Using technology in diabetes education reduces care inequities, increases the efficacy of educational initiatives, and improves accessibility. Technology as a means of diabetes education is supported by the broad use of telehealth and digital platforms by both patients and health care professionals [35]. Patients and health care professionals have accepted the use of telemedicine and other digital tools to educate people about diabetes. The COVID-19 pandemic has, in fact, significantly increased interest in and demand for telehealth-based diabetes education. The emergence and progress of digital health technologies have resulted in several modalities for engaging and educating patients through telephone or smartphone, video or audio, web, SMS text messaging, and mobile apps, as well as combinations of these methods [35].

In this study, participants are randomized into 3 intervention groups: an attention control group, a low-technology group, and a high-technology group. The purpose of this design is to evaluate the effectiveness of varying levels of technological integration in delivering medical nutrition therapy (MNT) and diabetes self-management education (DSME). All groups receive the same frequency and quality of remote education sessions. Both the low-technology and high-technology groups receive bidirectional SMS text messaging to facilitate timely communication with a health coach. In contrast, the attention control group does not receive any text-based communication or access to a health coach. Additionally, the high-technology group receives weekly diabetes-related educational materials via multiple channels (email and text), regular text reminders (eg, to read content, track health data, and stay motivated), and access to a telehealth platform. The low-technology group receives only 1 weekly email with diabetes-related educational materials. The attention control group does not receive any supplementary educational content or access to technology-based support resources.

The aim of this study is to implement and evaluate the impact of different levels of technology on the delivery of MNT and DSME among persons with mobility disability and T2D. We hypothesize that participants in the high-technology group will have improved self-management behaviors and better glycated hemoglobin (HbA_1c_) reduction compared to their counterparts in the low-technology and the attention control groups.

## Methods

### Study Design

This study, called YumABLE, started in June 2024, when recruitment began, and is anticipated to end by fall 2025. The study is a 3-arm randomized controlled trial for evaluating the delivery of a 6-month intervention, which involves telecoaching, diabetes educational content, and technology access, to 90 individuals with diabetes and physical disabilities. The 3 intervention groups are a high-technology group, a low-technology group, and an attention control group. Study participants are adults aged 18 to 65 years with a permanent disability and T2D. All study groups receive an initial 60-minute 1:1 assessment, 4-weekly remote DSME classes, a make-up class option, and a follow-up class 3 months after the last DSME class. Each of the weekly remote classes as well as the follow-up classes are 90 minutes in length. The 3-month follow-up remote class is 60 minutes in length. DSME classes are organized into cohorts, with each cohort consisting of up to 10 participants. DSME classes and education will be of the same quality and quantity for all participants regardless of study group.

**Figure 1:**
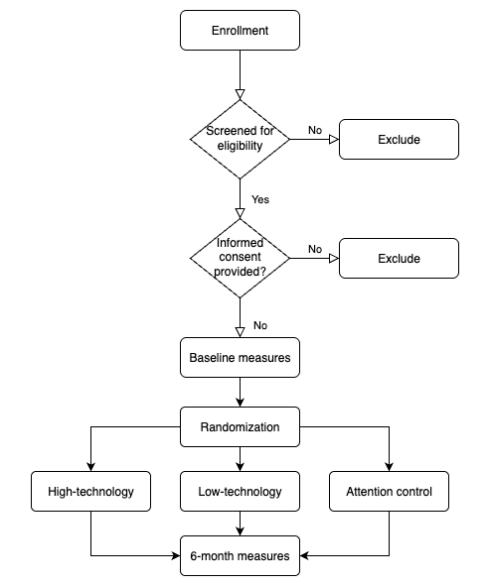
Flowchart of the protocol.

The difference among study groups is that the high-technology intervention group receives weekly diabetes-related materials delivered through multiple modalities (email, text, and telehealth platform), regular text reminders (to read the material, track health data, motivation, etc), and bidirectional SMS text messaging to enable timely communications with the certified diabetes care and education specialist (CDCES). Participants also have access to the telehealth platform. The low-technology intervention group receives 1 weekly email containing diabetes-related materials and bidirectional email communication with the CDCES. It does not have access to the telehealth platform. The attention control group, the “no technology” group, is only provided with DSME classes and has no technology support. It is not provided with any additional related content or access to technology-based support resources. Outcome measures are assessed at baseline and 6 months (Figure 1), with data collected from all intervention groups. Four weeks is considered a month in this study, making the study duration 24 weeks. Study activities are primarily conducted on the web nationally in the United States.

### Study Population

The American Diabetes Association defines a diagnosis of T2D as an HbA_1c_ level of 6.5% or higher, reflecting average blood glucose levels over the past 2 to 3 months. Likewise, the National Health and Nutrition Examination Survey’s Physical Functioning Survey will be used to identify permanent physical impairments. The participants consist of adults aged 18 to 65 years.

### Inclusion Criteria

Eligible participants must meet the following inclusion criteria: (1) have been diagnosed with T2D; (2) be aged 18 to 65 years; (3) be living with a permanent physical disability such as spinal cord injury, spina bifida, multiple sclerosis, or stroke; (4) have the ability to converse in and read English; (5) have a smartphone or computer available that can run apps; and (6) have Internet connection capabilities.

### Exclusion Criteria

Exclusion criteria were (1) current enrollment in any diabetes-related intervention, (2) a present or soon-planned pregnancy, (3) a major heart attack or heart surgery in the past 12 months, and (4) having undergone dialysis, kidney transplant, or a kidney surgery in the past 12 months.

### Recruitment

Participants are recruited digitally from the United States. Information about the study is shared through the National Center on Health, Physical Activity and Disability website and their affiliated social media outlets. Participants are additionally recruited from a database containing information on previous diabetes research studies with similar inclusion criteria. Emails are sent to these potential participants, providing details about the YumABLE program, and including a link for them to complete the study screening process and consent if they are eligible. During the screening process, participants are asked to self-report their HbA_1c_ levels to confirm their diabetes status. While many reported HbA_1c_ levels consistent with a diabetes diagnosis, baseline HbA_1c_ testing conducted by a contracted third party showed that some participants had lower levels, indicating prediabetes or normal glycemic status. These individuals were not excluded from the study, as it is possible that their HbA_1c_ levels had decreased since their previously reported values. Additionally, some participants, due to mobility challenges or limited access to the contracted laboratory, could not complete their baseline HbA_1c_ test. We accepted these individuals’ self-reported HbA_1c_ levels as measured during recent visits to their health care provider (within 3 months of enrollment in the study). Eligible participants who pass the screening process must sign an informed consent form and complete baseline surveys.

Upon enrollment in the study, participants receive an automated email containing a HIPAA (Health Insurance Portability and Accountability Act)-compliant REDCap (Research Electronic Data Capture) link to access a survey packet. REDCap is a secure program developed collaboratively by Vanderbilt University and the National Institutes of Health National Center for Research Resources [36]. A total of 28 participants have been recruited. The survey packet includes a demographic survey and primary outcome measures detailed in Table 1.

**Table 1:** Outcome measures and assessment timeline.

Variable		Instrument	Time point
**Primary outcome measures**			
	Diabetes quality of life	Diabetes Quality of Life Measure	Baseline, postintervention
	Psychological distress	Diabetes Distress Scale	Baseline, postintervention
	Self-efficacy	Diabetes Empowerment Scale	Baseline, postintervention
**Secondary outcome measure**			
	Glycemic management	HbA_1c_^a^	Baseline, postintervention
**Other prespecified outcome measures**			
	Physical activity	Godin Leisure-Time Exercise Questionnaire	Baseline, postintervention
	Dietary intake	The UK Diabetes and Diet Questionnaire	Baseline, postintervention
	Medication adherence	Medication Adherence Rating Scale	Baseline, postintervention
	Health dashboard usability	System Usability Scale	Baseline, postintervention

^a^HbA_1c_: glycated hemoglobin.

After participants receive the initial survey packet, a member of the research team calls the participants within a week. The purpose of this call is to welcome participants into the study and go over study components. During the call, the research personnel informed participants that they will receive a laboratory requisition in their mail within a week, allowing them to visit a third-party clinical testing company location to have their blood glucose tested at no cost. The research staff also lets participants know that they will be randomized into 1 of the 3 intervention groups. Participants were not informed about which intervention was the primary focus or “intervention of interest” versus the comparator groups.

### Randomization

After completing baseline surveys, participants are randomly assigned to 1 of 3 groups: the high-technology, low-technology, or attention control groups. The randomization sequence was created and stored in REDCap, automatically assigning participants to their respective groups with a 1:1:1 allocation ratio. The random allocation sequence was generated by study personnel using REDCap. Trained study staff enrolled participants and assigned them to intervention groups through the REDCap randomization module, which concealed the allocation sequence until assignment.

### Sample Size

In total, 90 participants will be enrolled, divided into 3 groups of 30 participants each (low-technology, high-technology, and attention control). The sample size is determined based on the primary use of analysis of covariance and uses a 2-sided test with a type I error rate of 0.05. An intention-to-treat analysis and multiple imputations are incorporated. Given a minimum correlation of 0.7 between the baseline and follow-up outcomes, there will be an 80% chance of detecting an effect size of 0.65.

### Intervention

The intervention is described in accordance with the TIDieR (Template for Intervention Description and Replication) checklist and guide [37]. Multimedia Appendix 1 shows the TIDieR checklist. As applicable, this protocol has been developed in accordance with the JMIR CONSORT-EHEALTH (Consolidated Standards of Reporting Trials of Electronic and Mobile Health Applications and Online Telehealth) checklist [38]. Multimedia Appendix 2 shows the JMIR CONSORT-EHEALTH checklist.

### Name and Rationale

The intervention is called YumABLE, and it addresses the effectiveness of using technology to deliver DSME for this target group. The name “YumABLE” reflects the program’s goal of empowering individuals with permanent physical disabilities to be “able” to manage their diabetes effectively, without limitations imposed by their disability. The key components of the intervention include a group class led by a single CDCES, ensuring a personalized experience for participants.

### Materials

Informational materials are distributed during the intervention. Informational materials consist of weekly resources on topics that include nutrition, meal plan methods, shopping tips, and a roadmap to managing diabetes, all provided by the CDCES.

### Procedures

Following randomization, the CDCES conducts a 60-minute one-on-one assessment with each participant via telephone. Subsequently, participants are assigned to a cohort for the 4 weekly 90-minute DSME group classes. Four optional 90-minute make-up classes are available along with a 60-minute follow-up session conducted 3 months after the final DSME coaching session. The intervention arms and treatment are summarized in Table 2.

**Table 2:** Study arms and intervention components.

Participant group or arm	Intervention or treatment
High-technology intervention group	Receive an initial 60-minute 1:1 call with the CDCES^a^. Receive 4 weekly 90-minute remote DSME^b^ classes with the option to make up any missed class. Receive one 60-minute follow-up class 3 months after their last DSME class. Weekly diabetes-related materials delivered through multiple modalities (email, text, and telehealth platform), regular text reminders (to read material, track health data, motivation, etc), bidirectional text messaging to enable timely communications with the health coach. Have access to the telehealth platform.
Low-technology intervention group	Receive an initial 60-minute 1:1 call with the CDCES. Receive 4 weekly 90-minute remote DSME classes with the option to make up any missed class. Receive one 60-minute follow-up class 3 months after their last DSME class. Receive 1 weekly email containing diabetes-related materials, bidirectional email communication with the CDCES. Have no access to the telehealth platform.
Attention control group	Receive an initial 60-minute 1:1 call with the CDCES. Receive 4 weekly 90-minute remote DSME classes and with the option to make up any missed class. Receive one 60-minute follow-up class 3 months after their last DSME class. Not be provided with any additional related content or access to technology-based support resources. Have no access to the telehealth platform.

^a^CDCES: certified diabetes care and education specialist.

^b^DSME: diabetes self-management education.

### Intervention Provider

A single CDCES, a board-certified registered nurse with over 15 years of clinical care, education, and health promotion experience, delivers all 4 remote DSME classes, encouraging active participation and reflection. Weekly goals are established and discussed, and troubleshooting is reviewed. Motivational interviewing promotes behavior change and sets personal health goals related to diabetes. The 4 DSME classes included nutritional management and diet, physical activity, medication adherence, and stress management components.

### Modes of Delivery

The classes are conducted remotely. The intervention consists of using pop-up quizzes or polls within each class session, having open discussions, and following up with participants, with communication based on their randomization group.

### Location

The intervention is conducted via video teleconferencing, providing an easily accessible platform for participants. This approach was particularly beneficial for individuals with physical disabilities, as it eliminated the need for travel to a physical location.

### Frequency and Duration

The intervention is for 6 months and includes 4 scheduled classes held consecutively over 4 weeks via video teleconferencing, 4 make-up classes for participants unable to attend an earlier class, and 1 follow-up class. Ideally, each participant would attend 5 sessions during this 6-month period.

### Tailoring and Modification

The intervention includes 3 participants with visual impairments; however, no changes are made to the intervention itself. Instead, materials are adapted to meet participants’ needs. Adaptations include providing the recorded class presentations with audio to participants who request it, weekly resources provided in larger text, and CDCES providing visual cues of the slides during the class to ensure that all participants understand what is being presented. Additionally, the video teleconferencing platform included accessibility features, such as captions for participants with impaired hearing, to ensure that they could fully participate.

### Ethical Considerations

The study was approved by the institutional review board (IRB) of the University of Alabama at Birmingham and is registered on ClinicalTrials.gov (NCT06049225). Informed consent from participants is required for participation in this study. Any modifications to the protocol will be communicated to all relevant stakeholders. The full trial protocol is available upon request from the corresponding author. The consent process is entirely web-based and initiated through predetermined screening questionnaires on the landing website and the REDCap link. Participants are informed of the purpose of the study, procedures, benefits, risks, and their right to withdraw from the study without any penalty. Once a participant is determined to be eligible through the web-based process, they receive an electronic consent form for completion. Access to records of the consent forms is limited to only approved study personnel. Participants’ data will be deidentified before data analysis. Participants are assigned unique IDs instead of their names or identifiable information. Data are stored on the University of Alabama at Birmingham’s secured password-protected servers, with access restricted to study personnel only. Participants are compensated with two US $50 e-gift cards, one for baseline and one for 6-month follow-up. In addition, participants who participate in qualitative interviews are compensated with an additional US $50. The compensation is to acknowledge participants’ time and efforts without creating undue influence.

### Outcomes

#### Overview

All primary and other prespecified outcome measures are collected and stored through electronic surveys delivered by REDCap. The validated questionnaire packet is electronically delivered to participants at baseline and 6 months. Baseline and postintervention assessments will evaluate both primary and secondary outcome measures. Table 1 outlines all outcome measures and their validated instruments for data collection. HbA_1c_ results are transmitted from the testing company to study personnel and inputted directly into REDCap. Similarly, if participants self-report their HbA_1c_ results, study personnel will input them directly into REDCap. To maximize participant retention, reminder emails are regularly sent. Additionally, detailed notes are recorded for participants who discontinue the study.

#### Primary Outcome Measures

##### Quality of Life

Participants will fill out the Diabetes Quality of Life Measure, a 15-item questionnaire that explores perceptions regarding one’s capacity to manage diabetes alongside other aspects of life [39].

##### Psychological Distress

The Diabetes Distress Scale, a 17-item questionnaire evaluating emotional distress associated with diabetes, will be used to measure participants’ psychological distress. This scale has demonstrated validity and reliability in measuring diabetes-related emotional distress [40,41].

##### Self-Efficacy

Participants’ psychosocial self-efficacy in diabetes management is assessed using the Diabetes Empowerment Scale Short Form. This 8-item questionnaire explores attitudes concerning diabetes and the capability to effectively manage the condition. The Diabetes Empowerment Scale Short Form has exhibited validity and reliability as a measure of psychosocial self-efficacy [42].

#### Secondary Outcome Measure: HbA1c Levels

HbA_1c_ is measured using an HbA_1c_ test. HbA_1c_ results are transmitted from the testing company to study personnel and inputted directly into REDCap. Similarly, if participants self-report their HbA_1c_ results, study personnel will input them directly into REDCap. HbA_1c_ is measured both at baseline and at 6 months. The HbA_1c_ is a widely recognized test to assess the effectiveness of diabetes management strategies.

#### Other Prespecified Outcome Measures

##### Physical Activity

Physical activity is assessed through the Godin Leisure-Time Exercise Questionnaire. This brief questionnaire comprises 3 questions concerning strenuous, moderate, and light exercises undertaken within the past 7 days [43].

##### Dietary Intake

Participants will complete the UK Diabetes and Diet Questionnaire to evaluate nutrition and dietary behaviors over the past month. This questionnaire features foods commonly found in the United Kingdom. Therefore, to ensure clarity for participants from the United States, any UK-specific food item will be substituted with its equivalent US counterparts [44].

##### Medication Adherence

Participants will complete the Medication Adherence Rating Scale to assess their medication adherence. This general 10-item binary questionnaire asks participants about regular medication intake [45].

##### Health Dashboard Usability

To evaluate the usability of the telehealth dashboard, participants will complete the System Usability Scale. This Likert scale consists of items assessing the dashboard’s effectiveness (ability to accomplish tasks), efficiency (level of dashboard use), and satisfaction (subjective reactions to the dashboard) [46,47].

### Data Management

All data gathered throughout the study will be entered directly into the REDCap system. Upon enrollment, participants will be assigned a unique subject number for identification purposes within the survey. Access to the REDCap program, where all data will be securely stored, will be limited to authorized study personnel.

The principal investigator (MT) and primary statistician (KRH) remain blinded to the randomization of participants across the study arms throughout the protocol. Other designated study staff will have access to this information for specific tasks such as recruitment, obtaining consent, conducting orientation and welcome calls, coaching sessions, and managing the shipment of intervention packages.

We will also perform data verification, including confirming the completion of surveys, questionnaires, and the accurate recording of HbA_1c_ values. Given that this project is a pilot feasibility and efficacy study with a duration of 6 months and is deemed to have minimal risks, a data monitoring committee is unnecessary. The principal investigator (MT) holds responsibility for maintaining protocol fidelity and overseeing data collection procedures throughout the study period.

### Analyses

#### Statistical Analysis

Descriptive statistics will be prepared to show the sample statistics and analyzed separately for baseline and 6-month postproject. Paired 2-tailed *t* tests and chi-square tests will be used to assess the change in continuous and discrete outcomes from baseline to 6 months for each arm. Furthermore, within-group differences will be assessed using 2-tailed *t* tests (paired and unpaired) and chi-square tests.

Multivariate linear mixed models will be used to assess repeated measures with continuous outcomes, assess the joint effects of baseline measures on 6-month outcomes, and account for the correlation between repeated measures. Quality control analyses will be performed, including assumption checking, estimating descriptive statistics of participant attrition and overall instrument completion, and investigating missingness patterns. Key variables at baseline will be examined to evaluate whether there is any systematic variation between the study groups. A participant who no longer participates in study activities will be considered lost to follow-up. App analytics on key performance indicators will be used for participants who are lost to follow-up. We anticipate the loss to follow-up rate to be approximately 20%. The primary analysis will follow the intention-to-treat principle, including all participants as originally assigned regardless of adherence. Secondary or compliance analyses will be considered if appropriate.

All qualitative data collected in the study will be content analyzed using NVivo (Lumivero), a qualitative data analysis software [48], including qualitative data collected for the feasibility, acceptability, and fidelity of interviews and meeting notes. The steps for content analysis include (1) transcribing audio recordings, (2) checking transcription against recordings for accuracy, (3) developing coding categories, (4) assigning codes to text, and (5) reviewing coded data to identify themes across the dataset.

#### Qualitative Analysis

Moreover, the study will include qualitative interviews conducted after the intervention to evaluate the program thoroughly. These interviews aim to assess the intervention’s effectiveness as a feasible approach for managing diabetes. Multimedia Appendix 3 shows the interview guide. The interviews will be transcribed and analyzed using an inductive content analysis method as described by Elo and Kyngäs [49]. The analysis will be performed in 3 phases. The first phase is open coding, followed by creating categories and abstraction [49]. NVivo software will be used to facilitate the organization and analysis of the qualitative data.

### Data Monitoring

Any personal information collected during the study, including demographic details and other sensitive data, will be gathered and stored in compliance with the HIPAA regulations using the secure REDCap platform. Access to this confidential information will be limited to authorized study staff who have received approvals. The storage and handling of personal data will adhere to the guidelines, oversight provided by the university’s IRB, and the policies established by affiliated entities. The laboratory testing service will manage the collection and processing of blood samples for HbA_1c_ testing. Study personnel will not handle or have access to the blood samples at the testing laboratory.

### Harms

This study will track and evaluate all adverse events. These events will be assessed for their severity and potential causality. Any identified adverse events will be reported to the IRB and other pertinent regulatory bodies, as required by the study protocols and ethical guidelines.

### Auditing

The study protocol will undergo regular evaluations at specified intervals, such as weekly, monthly, or quarterly, to ensure adherence to the study’s design and consistent implementation. Auditing procedures will encompass various elements, including checklists, coaching call logs, audio recordings or coaching calls, content resource banks, telehealth platform reviews, and event logs; review of participant food, physical activity, medication, glucose entries, and time spent on the platform; and team meetings to discuss participant progress and protocol adherence.

## Results

We hypothesize that participants in the high- or low-technology intervention groups will show improvements in self-management behaviors related to T2D and self-efficacy compared to the control group at the 6-month follow-up. Again, we hypothesize that the high-technology intervention arm will result in better outcomes regarding reductions in HbA_1c_ than the low-technology and control arms. Additionally, we also hypothesize that the YumABLE intervention is feasible and acceptable to adults with permanent physical disability and T2D and that fidelity to the program will be maintained.

The development of the telehealth platform, which includes the dashboard and educational content, along with the creation of the landing website, has been completed by the study’s technical support team. In addition, research personnel have developed the database using REDCap to serve as the storage for data and communication tools such as emails and text notifications. The study began in June 2024. Since June 2024, intervention materials have been finalized, the IRB submissions have been completed, and participant recruitment began. In total, 28 participants have been enrolled and received the MNT and DSME remote class. Recruitment has ended, and we anticipate completing data collection by August 2025. Make-up classes were delivered to participants in any of the 3 cohorts between November 2024 and December 2024. The final 3-month follow-up classes were held for each cohort 3 months after the first class and were completed in February 2025. Data will be analyzed in fall 2025. Manuscript preparation and dissemination of study results are planned for spring 2026.

## Discussion

Previous research has showed that comprehensive diabetes education is required to equip patients with the necessary self-management skills needed to manage the condition. Likewise, diabetes education has been found to improve patient knowledge, improve diabetes control, and delay complications [50-56]. The American Diabetes Association has pointed diabetes education as the cornerstone therapy for the patient with diabetes [54].

Numerous publications have highlighted the importance of technology in diabetes care and management [34,35,57]. Sharma et al [57] posit that technology use in providing diabetes education leads to improved patient access and clinical outcomes, reduces the cost of care, and addresses gaps in care. Patients with diabetes who get live telephone coaching for a specified duration can gain advantages from consistent health status monitoring, leading to enhanced self-efficacy, improved health outcomes, and decreased hospitalization rates, surgical expenses, and total health care expenditures [58-61]. The reduction of HbA_1c_ levels has been a notable benefit achieved by telephonic support [60].

Likewise, web-based education is an effective tool for promoting diabetes self-management. Some websites exclusively provide diabetes-related content and resources, while others include an opportunity to interact with a coach [62]. Other websites provide digital record-keeping tools that enable patients to track and monitor their food and physical activity as well as generate reports or logs [63]. Moreover, other websites include blogs, healthful recipes, menus, and holiday meal planning assistance [57]. Some clinical web-based supportive educational tools include remote chatting, discussion forums, emailing choices, and the ability to contact a live diabetes coach [57]. Indeed, web-based educational interventions have shown promise in supporting diabetes self-management. Such digital training approaches have been linked to enhanced treatment adherence and improved health indicators, highlighting their potential as effective tools in early diabetes care [63].

Given that self-management is crucial in managing T2D and also considering that persons with disabilities face barriers that prevent them from successfully managing their diabetes, a study looking to create an available, inclusive, and accessible diabetes management program for people with disabilities is important. To the best of our knowledge, there is no existing program in the United States explicitly providing remote DSME and MNT tailored for persons living with disabilities.

This study has limitations. Factors such as the supportive group setting, the instructor, or individual components of the program may influence health outcomes in different ways. To account for potential confounding factors, we have chosen a randomized design. Additionally, behavior changes, such as participating in an organized intervention, could contribute to improvements, including adopting healthier diets. As with any complex intervention, various factors may impact the outcomes. However, this reflects real-world clinical scenarios. Using a randomized design, the study aims to minimize the influence of confounding variables and provide more reliable findings. Additionally, several aspects of our trial design may pose potential limitations. First, participants with mobility limitations or accessibility issues may be unable to visit the contracted laboratory for their HbA_1c_ test and instead self-report their HbA_1c_ levels. These self-reported data may not be entirely accurate or up to date. Additionally, as the study is conducted remotely, some participants, particularly older individuals, may face technical challenges, such as difficulty navigating the platform or accessing the intervention materials.

That notwithstanding, this study has some strengths. To begin with, the methodological strengths of the study include the randomized controlled design, preregistration in a clinical trials registry, the inclusion of make-up and follow-up classes, and the use of both quantitative and qualitative measures. Another strength is a third-party testing company’s involvement in the HbA_1c_ tests, ensuring impartiality and reliability in the results.

The results from this study will expand our understanding of how technology-delivered behavioral interventions can effectively address the challenges of managing diabetes, particularly among individuals with permanent physical disabilities and their families. A combined approach of DSME and technology offers a promising, easily accessible, and engaging intervention that may serve as a practical pathway for improving diabetes management.

This study provides future directions. First, it can lead to more studies on how artificial intelligence could be used in the delivery of diabetes education among individuals with various forms of disabilities. In addition, the study can help provide insight regarding an effective approach to combine more than 1 technology in providing diabetes management education.

We intend to present study findings at diabetes, endocrinology, and digital health conferences and submit the data for publication in peer-reviewed open-access publications. In addition, we will consult with health care leaders and diabetes care teams within our health system on potential changes to diabetes education methods and digital intervention protocols.

In conclusion, this randomized controlled study, with an anticipated 90 participants, will explore the effects of combined DSME and technology among individuals with diabetes and a permanent physical disability. The primary aim is to assess how technology-enhanced DSME influences diabetes management. The findings from this intervention study could offer valuable insights for health care professionals, health educators, and public health personnel, informing future strategies for managing diabetes in individuals with physical disabilities and more broadly among all persons living with T2D.

## Appendix

Multimedia Appendix 1 TIDieR checklist.

Multimedia Appendix 2 CONSORT-eHEALTH checklist (V 1.6.1).

Multimedia Appendix 3 Final interview guide.

Multimedia Appendix 4 Peer review report by National Institute on Disability, Independent Living, and Rehabilitation Research (NIDILRR), Administration for Community Living (National Institutes of Health, USA).

## Abbreviations

CDCES: certified diabetes care and education specialist
CONSORT-EHEALTH: Consolidated Standards of Reporting Trials of Electronic and Mobile Health Applications and Online Telehealth
DM: diabetes mellitus
DSME: diabetes self-management education
HbA1c: glycated hemoglobin A1c
HIPAA: Health Insurance Portability and Accountability Act
IRB: institutional review board
MNT: medical nutrition therapy
REDCap: Research Electronic Data Capture
TIDieR: Template for Intervention Description and Replication
T2D: type 2 diabetes

## Data Availability

The datasets generated or analyzed during this study are available from the corresponding author on reasonable request.
