# Supplementary material for: A Technology-Enhanced Medical Nutrition Therapy and Diabetes Self-Management Education for Adults With Disability and Type 2 Diabetes: Protocol for a Pilot and Feasibility Randomized Controlled Trial
Source: JMIR Res Protoc. 2025 Sep 26;14:e71495. doi: 10.2196/71495 (PMC12514415; doi:10.2196/71495)
Supplement: Multimedia Appendix 3 [file resprot_v14i1e71495_app3.pdf]

Section 1: Background and Initial Experience with Prediabetes

1. **Can you tell me about when you first found out you had prediabetes?**
   - o How did you feel when you received the diagnosis?
   - o What did you know or understand about prediabetes at that time?

2. **What were the first steps you took after learning you had prediabetes?**
   - o Did you seek any information or support? If yes, where/from whom?
   - o Were you given specific recommendations or advice by a healthcare professional?

3. **Did you make any lifestyle changes during this time?**
   - o If yes, what kind of changes did you make (e.g., diet, exercise, weight management)?
   - o Were these changes easy or difficult for you to implement? Why?

Section 2: Transition from Prediabetes to Diabetes

4. **Can you describe your transition from being diagnosed with prediabetes to being diagnosed with diabetes?**
   - o How long after the prediabetes diagnosis were you diagnosed with diabetes?
   - o Were there any specific factors or events that contributed to this transition?

5. **How did you feel when you learned you now had diabetes?**
   - o What concerns or worries did you have at the time?
   - o Did your understanding of diabetes change after the diagnosis?

Section 3: Experiences with the Study Intervention

6. **Can you describe your overall experience with the diabetes management intervention offered in the study?**
   - o How did you feel about the program when you first started?
   - o Were your expectations met throughout the course of the intervention?

7. **Can you describe any positive changes or improvements as a result of participating in the study? (Yes/No) Please explain.**
   - o In what specific ways did the intervention affect your [health/behavior/skills]?

8. **Which aspects of the intervention were the most helpful to you?**
   - o Was there a particular component (e.g., educational sessions, coaching, tracking tools) that made a significant difference in your diabetes management?
   - o Can you share any specific examples of how these elements helped you?
   - o Did you notice any positive changes or improvements as a result of participating in the study? (Yes/No) Please explain.
   - o In what specific ways did the intervention affect your [health/behavior/skills]?

9. **In what ways Did the intervention change your attitude or beliefs about your ability to self-manage diabetes with other chronic conditions and a physical disability? If yes, in what ways?**
   - Have you made any changes to your daily behavior or habits as a result of the intervention? Please describe.
   - Have you seen any lasting effects from the intervention? If so, what are they?
10. **What factors in your life (e.g., work, family, personal circumstances) made it easier or harder to engage with the intervention?**

11. **How much time did you spend on the intervention each week on average?**

12. **What aspects of the intervention that you found challenging or unhelpful?**
    - What were the barriers, if any, to participating fully in the intervention?
    - Did you encounter any difficulties with the program format, materials, or frequency of sessions?
13. **How did you feel about participating in group sessions via Zoom?**
    - Was the virtual format convenient for you, or did it present any challenges (e.g., technical difficulties, distractions)?
    - Did you feel comfortable sharing your experiences in a virtual group setting?

14. **What was the group dynamic like during the Zoom sessions?**
    - Did you feel connected with other participants?
    - Was there a sense of community and mutual support, or did the virtual format make it harder to connect?

15. **How did the facilitator manage the Zoom sessions?**
    - Did you feel that the sessions were well-organized and engaging?
    - Were the facilitators effective in addressing participants' questions or concerns?

16. **How did the support provided by the intervention (e.g., Study staff, educational resources, or peer groups) impact your management of diabetes?**
    - Did it change your approach to diet, exercise, medication, or other lifestyle factors?
    - How did the level of support compare to what you were receiving before joining the study?

<u>Section 4: Reflections on What Went Well and What Could Have Been Improved</u>

17. **Looking back, what aspects of the diabetes management program went well?**
    - Were there any turning points or helpful interventions that stood out?
    - What do you think contributed most to your progress in managing diabetes?

18. **What could have been done better or differently to enhance the program?**
    - Were there any gaps in the support or resources provided?
    - Do you have suggestions for changes that would make the program more effective for future participants?

19. **If you could design an ideal diabetes management program, what would it look like?**
    - What components would you include that were not part of this study?
    - How would you improve participant engagement/interaction and outcomes?

20. Would you recommend this intervention to others facing similar challenges? (Yes/No/Maybe) Why or why not?
