# Supplementary material for: A Technology-Enhanced Medical Nutrition Therapy and Diabetes Self-Management Education for Adults With Disability and Type 2 Diabetes: Protocol for a Pilot and Feasibility Randomized Controlled Trial
Source: JMIR Res Protoc. 2025 Sep 26;14:e71495. doi: 10.2196/71495 (PMC12514415; doi:10.2196/71495)
Supplement: Multimedia Appendix 4 [file resprot_v14i1e71495_app4.pdf]

Improving the Lives of Older Adults and People with Disabilities
Through Services, Research, and Education

August 12, 2022

Shireen Abdullah
603 Munger Avenue
#100
Dallas, TX 75202-1840

Applicant Name: ***Kamin Consulting, Inc.***
Application Number: ***BISB22000200***

Dear Ms. Abdullah:

Congratulations! Your application to the National Institute on Disability, Independent Living, and Rehabilitation Research (NIDILRR) for consideration under the Fiscal Year 2022 ***Small Business Innovation Research (SBIR) Phase II*** grant competition has been selected for funding.

Each application was objectively reviewed, discussed, and scored by a peer review panel. Panel members evaluated applications according to the criteria published in the funding opportunity announcement. I am enclosing a copy of the comment sheets prepared by the peer reviewers. If you wish to discuss the peer review process, the reviewers' comments or any related matters pertaining to your application or NIDILRR's SBIR Program, please contact Brian Bard at Brian.Bard@acl.hhs.gov or by phone at (202) 795-7298.

Please note that the start date for the grant described in this letter is September 1, 2022.

Thank you for your interest in the SBIR Grants. I look forward to your continued interest in NIDILRR grant programs.

Sincerely,

Anjali J. Forber-Pratt

Anjali J. Forber-Pratt, PhD
Director
National Institute on Disability, Independent Living, and Rehabilitation Research

Enclosures

Average Score: 87.80

Application Number: BISB22000200
Application Name: Kamin Consulting Inc.
State: TX City: DALLAS

Criteria Name (Max Score)

1. Importance of the Problem (20 Points)
2. Quality of Project Design (50 Points)
3. Project Staff (15 Points)
4. Adequacy and Reasonableness of the Budget (5 Points)
5. Adequacy and Accessibility of Resources (10 Points)

TOTAL: 100

Summary of Panel Discussion

Strength:

Overall, reviewers agreed that the applicant adequately explained the needs of individuals with Type-2 diabetes and disabilities and on the prevalence of diabetes and the challenges faced by people with disabilities. The applicant describes current and related educational programs and some reviewers commented that the literature review supports the proposed methods. The project design is derived from a 6-week pilot study that suggests the product is effective. Reviewers agreed that the use of health coaching is appropriate and innovative. The application includes strong work, commercialization and technology accessibility plans. All reviewers agreed that the project staff have appropriate experience and expertise in multiple areas and that the project staff are qualified and well trained. The staff includes individuals from members of underrepresented groups and the applicant describes a commitment to encouraging further employment of individuals from underrepresented groups. All reviewers agreed that the project costs are reasonable, and they align well with the project goal. A detailed budget narrative is provided for each year and budget items are well justified. A good discussion on accessibility is included. The applicant, a minority-owned organization, has sufficient resources, equipment, and strong partnerships with the University of Alabama, Birmingham and Lakeshore Foundation to support project activities.

Weakness:

Reviewers agreed that the specific target population is not clearly defined. People with disabilities are frequently discussed as a homogeneous group without the acknowledgement that they have unique needs, often based upon their disability type. Some reviewers agreed that a more systematic and comprehensive review of current and relevant literature would have strengthened the proposal. Some reviewers had concerns about the methodological approach. All reviewers noted that Blood Glucose Monitors should be incorporated into the product development. Some reviewers were concerned with the lack of clarity on how the overall goal of the project, to create a reimbursable and effective Type II diabetes management solution to meet the need of people with disabilities will be achieved. Information is not provided on how participants will be recruited for the product evaluation. There is limited discussion on how input from individuals with disabilities and other key stakeholders will be collected during Phase II; interview procedures are not clearly described. Some reviewers commented that no project staff on the team has expertise in limited mobility and that the applicant did not include a job description for the software developer who will be a key staff member. Some reviewers pointed out that a subcontractor budget is not included in the budget justification or narrative.

General Feedback:

None noted.

Technical Review Form : 1

Criteria Name (Max Score) : Actual Score

1. Importance of the Problem (20 Points) : 18
2. Quality of Project Design (50 Points) : 48
3. Project Staff (15 Points) : 14
4. Adequacy and Reasonableness of the Budget (5 Points) : 5
5. Adequacy and Accessibility of Resources (10 Points) : 10

TOTAL: 95 / 100

Rationale

Scoring Criteria

Criterion 1: Importance of the Problem

Strength:

Page: 1-7

Applicant clearly describes the need for a tele-health diabetes management and education program for individuals with disabilities.

Page: 1-8

The proposed activities address a significant need of individuals with disabilities.

Page: 8-9

Proposed activities will have a direct impact on the health and independent living of individuals with disabilities who have diabetes.

Weakness:

Page: 1-9

The applicant does not directly state the target population.

Criterion 2: Quality of Project Design

Strength:

Page: 17

Project builds upon phase 1 prototype.

Page: 18

Development of the User Journey Map as a measure of user experience.

Page: 19

Table 1 outlining the technology accessibility plan with options for different disabilities.

Page: 28

Input from individuals with disabilities is obtained and will inform proposed development activities.

Page: 9-15

The proposed project includes a thorough high-quality review of the relevant literature, identification of barriers to individuals with disabilities accessing other health related educational sites, and a plan for project implementation.

Page: 9-28

The design of the project is appropriate and is likely to successfully address the needs of the target population.

Weakness:

Page: 17-28

Use of integration of continuous glucose monitoring should be addressed. Such state-of-the-art technologies should be incorporated as a option for participants.

Page: 28

How the input from disabilities and key stakeholders will be used to inform later stages of the project is needed.

Criterion 3: Project Staff

Strength:

Page: 28

Project work plan outlines key tasks, measurable outcomes, and responsible personnel for each task.

Page: 29

Applicant outlined history of employment from traditionally underrepresented groups.

Page: 29-32, appendices

Key personnel and staff have appropriate training and experience to conduct all proposed activities.

Page: 30-32

Commitment of staff time is adequate to accomplish all proposed activities.

Weakness:

Page: 32

Project hinges on, and allots for significant time for, an unnamed software developer. The tasks are critical, time sensitive, and the large amount of time allocated to an unnamed subcontractor should be addressed with a job description or list of skill sets needed.

Criterion 4: Adequacy and Reasonableness of the Budget

Strength:

Page: 33-37, appendices

The costs are reasonable in relation to the proposed project activities.

Page: 33-37, appendices

The budget for the project, including subcontractors, is justified to support the proposed project activities.

Weakness:

None

Criterion 5: Adequacy and Accessibility of Resources

Strength:

Page: 40

The applicant is committed to providing adequate facilities, equipment, and administrative support as appropriate.

Page: 40

The applicant outlines the accessibility of resources.

Weakness:

None

Technical Review Form : 2

Criteria Name (Max Score) : Actual Score

1. Importance of the Problem (20 Points) : 10
2. Quality of Project Design (50 Points) : 40
3. Project Staff (15 Points) : 13
4. Adequacy and Reasonableness of the Budget (5 Points) : 5
5. Adequacy and Accessibility of Resources (10 Points) : 10

TOTAL: 78 / 100

Rationale

Scoring Criteria

Criterion 1: Importance of the Problem

Strength:

None

Weakness:

<ins>Page: 24-28</ins>

This project is for the development of a product that will meet the needs of people with diabetes. While that is indeed a disabling condition, it is not clear to me how other products on the market don't also meet the needs of people with disabilities such as diabetes. Are the authors also going to address those with additional physical disabilities? Exactly how does this product potentially help people with disabilities in a different way than the products currently on the market?

Criterion 2: Quality of Project Design

Strength:

None

Weakness:

Page: 30-52

My primary issue with this proposal is the lack of clarity about what current products on the marketplace to address this issue lack in terms of assisting the target population of people with diabetes....unless there is an additional criteria (which is not outlined) for a secondary physical disability as well. Other than the current products are more expensive and rely on mobile apps (page 59), I cannot determine how this product would enhance the options available to people with diabetes. Also, the next objectives outlined on Pages 58-59 are not clear to me at all. Additionally, on page 23 it states that the goal of this project is to create a reimbursable....Type 2 diabetes management solution to meet the needs of people with diabetes. I cannot find anywhere else in the proposal where it is clearly explained how this will be accomplished, and from what funders reimbursement would happen.

Criterion 3: Project Staff

Strength:

None

Weakness:

Page: 140-167

Cannot find anyone with expertise in physical disabilities on the team. Also there is no sub-contractor budget.

Criterion 4: Adequacy and Reasonableness of the Budget

Strength:

Page: 177-184

7% fee, 10% indirect. Salaries and benefits are in line with market rates.

Weakness:

None

Criterion 5: Adequacy and Accessibility of Resources

Strength:

Page: 61-63

Affiliated with University of Alabama at Birmingham, and Lakeshore Foundation. Dallas offices are minimal with 200 sq ft of office space.

Weakness:

None

Technical Review Form : 3

Criteria Name (Max Score) : Actual Score

1. Importance of the Problem (20 Points) : 15
2. Quality of Project Design (50 Points) : 40
3. Project Staff (15 Points) : 13
4. Adequacy and Reasonableness of the Budget (5 Points) : 4
5. Adequacy and Accessibility of Resources (10 Points) : 10

TOTAL: 82 / 100

Rationale

Scoring Criteria

Criterion 1: Importance of the Problem

Strength:

Page: 1-4

The application includes a carefully referenced discussion of the challenges faced by individuals with Type 2 Diabetes and the efficacy of existing diabetes prevention and management programs.

Page: 5-6

The application describes the accomplishments of Phase I and the ways that the proposed product could support individuals with disabilities.

Weakness:

Page: 1-6

The application refers to individuals with disabilities as if they are one homogeneous group when in fact there is a wide range of disabilities each with different barriers to diet and exercise. The application would have been strengthened if it had reviewed research relevant to individuals with impaired mobility since this is the proposed test group.

Criterion 2: Quality of Project Design

Strength:

Page: 12-13

The application reviews literature related to Medical Nutrition Therapy and its use with individuals with diabetes.

Page: 13

The application discusses other commercial diabetes management platforms and explains why these products do not meet access and inclusion criteria.

Page: 13-14

The application clearly describes the inclusion and exclusion criteria for the three groups in the randomized control pilot study. The intervention is well described.

Page: 26-27

The application includes a good description of how fidelity and efficacy will be measured and lays out the data management plan in detail.

Page: 28-29

The application presents a nicely developed work plan with details in Table on page 29.

Page: 34-38

The application includes a detailed commercialization plan that responds to the seven points required by the FOA.

Page: 7-8

The application discusses two Specific Aims of the SBIR II in detail. These Specific Aims are grounded in the findings from Phase I.

Page: 8

The application proposes to use User Centered Design and Lean Product Development methods for Aim 1 and a randomized control design for the Aim 2 pilot study. All proposed methodologies are appropriate to the development process.

Page: varies

The application's narrative makes it clear that the product designers are up-to-date on relevant literature and familiar with their competitors.

Weakness:

Page: NA

The application does not discuss the advances in Continuous Blood Glucose Monitors and how this technology could be integrated into and enhance the proposed product.

<u>Page: varies</u>

The application proposes a product/intervention that requires a dietician talk with the consumer every week for six months, but there is no analysis of how expensive the staffing will be or whether potential buyers will have that level of dietician time available.

<u>Page: varies</u>

The application does not provide any information about how individuals will be recruited for the RCT, whether they have used this recruitment method before and its success rate.

<u>Page: varies</u>

The application makes the assumption that a dietician will have the skills and training necessary to motivate and coach individuals to act differently with regard to food and exercise. There is no discussion about training or supervision for the dieticians.

Criterion 3: Project Staff

Strength:

Page: 29-30

The application provides a brief description of the diversity of staff at Kamin and their attempt to hire diverse individuals.

Page: 30-32

The application allocates staff time in a way that seems adequate with more time going to individuals in year two who will be actually implementing and testing the intervention.

Page: 30-32

The application bids potential staff who have the education and experience to develop the product and test it. This staff has deep knowledge about diabetes and diabetes education.

Weakness:

Page: NA

The application contains no information about experience and skill level that will be expected for the research assistant nor the amount of FTE allocated to this position.

Page: NA

The application does not describe any staff member or consultant who has experience with individuals with limited mobility issues.

Criterion 4: Adequacy and Reasonableness of the Budget

Strength:

Page: Budget Narrative

The application includes a budget narrative that includes line item budgets for the two years. Proposed costs are reasonable and well justified.

Weakness:

Page: Budget Narrative

The application does not include budgets for the subcontractors.

Criterion 5: Adequacy and Accessibility of Resources

Strength:

Page: 29-40

The application argues that adequate facilities and equipment are available for the staff of Kamin. All facilities and equipment are accessible to individuals with disabilities.

Weakness:

None

Technical Review Form : 4

Criteria Name (Max Score) : Actual Score

1. Importance of the Problem (20 Points) : 18
2. Quality of Project Design (50 Points) : 46
3. Project Staff (15 Points) : 14
4. Adequacy and Reasonableness of the Budget (5 Points) : 5
5. Adequacy and Accessibility of Resources (10 Points) : 10

TOTAL: 93 / 100

Rationale

Scoring Criteria

Criterion 1: Importance of the Problem

Strength:

Page: 1-9

The applicant clearly describes the need of people with disabilities. For example, the applicant documents that " Diabetes mellitus (DM) is one of the most common metabolic disorders affecting about 11.3% of the overall US adult population, which is about one in every ten people1. 38.0% of the US adult population is affected with prediabetes1." (1) The applicant clearly describes how the proposed project will address a significant need of the target population by developing " the high-fidelity functional telehealth platform (YumAble) for effectively delivering a Medical Nutrition Therapy (MNT) and Diabetes Self-Management Education (DSME) program for people with type-2 diabetes and disabilities." (8) The applicant provides a brief discussion on the potential impacts of the project on target population, that is, "Achieving the study aims will directly benefit people with physical disabilities in managing their diabetes. Successful completion of the second aim will create an advanced technology-backed medical nutrition therapy and diabetes self-management education program for people with disabilities, that will then be evidence-based and shall be commercially offered backed by insurance payment." (p. 9)

Weakness:

Page: 1-9

Target population (people with Type 2 diabetes, people with disabilities, adults with permanent impaired mobility, people with physical disabilities with diagnosed Type 2 Diabetes., people with physical disabilities?) is not clearly described. (iii).

Criterion 2: Quality of Project Design

Strength:

Page: 9-29

Three objectives are stated in measurable terms and they align well with the primary project goal. "The objectives: (1) completion of a fully functional telehealth platform; (2) development of content, scripts, infographics, and videos needed for full accessibility and inclusion; and (3) conducting a nationwide three-arm randomized controlled trial to establish effectiveness of our program, content, and technology." (iii. Abstract) A brief review of current and relevant literature is provided on self-management solutions for T2DM (9). The design of the project reflects up-to-date knowledge from research and effective practice. The applicant demonstrates awareness of current and related programs (4). For example, the applicant documents that "Several proven diabetes prevention and management programs exist, including CDC's Diabetes Prevention Program (now called Prevent T2) and Stanford's Diabetes Self-Management Program (DSMP)." (4) as well as current state of technology-mediated solutions for diabetes and medical nutrition therapy. (10-11). A detailed work plan is provided with key tasks, measurable outcomes, lead persons, and milestones for each year. (29). Input from individuals with disabilities and other key stakeholders were gathered during Phase I (28).

Weakness:

Page: 8-29

The goals, objectives, and specific aims are not well aligned with each other. For example, the goal of the proposed project is: "to create a reimbursable and effective Type 2 diabetes management solution to meet the needs of people with disabilities." (iii. Abstract). On p. 9, the applicant states "The primary objective of this project is to develop, and pilot test an accessible and inclusive medical nutrition therapy and diabetes self-management education program for people with physical disabilities with diagnosed Type 2 Diabetes." This objective is different from the three mentioned in the abstract. It is not clear how the specific aim aligns with the three objectives. (8-9) On p. 28, the applicant lists two goals: Goal 1: Develop high-fidelity fully functional prototype of the telehealth platform Measurable outcome: Functional and usable prototype. Goal 2: Completed pilot feasibility evaluation of the YumAble intervention." The proposal would benefit from a more comprehensive review of research literature. It is not clear how the 90 adults with T2DM and permanent impaired mobility will be recruited for the feasibility pilot study (20) Interview procedures are not clearly described. (26) There is limited discussion on how input from individuals with disabilities and other key stakeholders were gathered during Phase II.

Criterion 3: Project Staff

Strength:

Page: 30-31

The applicant adequately describes the project staff. The team is strong, and the members have relevant experiences and expertise to carry out the proposed project. For example, PI Abdullah " has 15 years of experience in management consulting and project management experience from the public and private sector and across a breadth of industries, including local government, utilities, and education." (30) Other key personnel also have relevant experiences to this project (digital health, nutrition science, intervention implementation and evaluation). (31)

Weakness:

Page: 30-31

No project staff on the team has expertise in mobility.

Criterion 4: Adequacy and Reasonableness of the Budget

Strength:

<u>Page: budget</u>

Costs are reasonable in relation to the project. (33) A detailed budget narrative is provided for each year and budget items are well justified.

Weakness:

None

Criterion 5: Adequacy and Accessibility of Resources

Strength:

Page: 39

The applicant states that the "project's activities will draw on the extensive and highly interrelated expertise available at Yumlish, University of Alabama at Birmingham, and Lakeshore Foundation." (38). Information on resources of Yumlish and Lakeshore Foundation are provided. Kamin Consulting has meeting space, videoconferencing equipment, etc. (39)

Weakness:

Page: None

No comment.

Technical Review Form : 5

Criteria Name (Max Score) : Actual Score

1. Importance of the Problem (20 Points) : 17
2. Quality of Project Design (50 Points) : 46
3. Project Staff (15 Points) : 14
4. Adequacy and Reasonableness of the Budget (5 Points) : 4
5. Adequacy and Accessibility of Resources (10 Points) : 10

TOTAL: 91 / 100

Rationale

Scoring Criteria

Criterion 1: Importance of the Problem

Strength:

Page: 1-9

- Applicant provides data on prevalence and impacts of diabetes, including the disparity of rates of individuals with disabilities diagnosed with diabetes compared to the general population, and discusses the risk of secondary disabilities due to diabetes, all that help to substantiate the need for product development for the target population, p. 1. - Substantiation is provided for the importance of nutrition and activity management to prevent and/or control Type II diabetes symptoms, p. 1-3. - In Phase I, the applicant trialed a diabetes therapy and management program for individuals with disabilities - apparently the first program for individuals with disabilities and diabetes - that resulted in useability scores of 68 and 81 (without outliers) that provides justification for a next phase of product development, p. 4-7. - Applicant appropriately seeks funding to 1) develop a platform for delivering the program; 2) conduct a randomized and controlled pilot study to determine the feasibility and acceptability of individuals with physical disabilities and diabetes, p. 7-9.

Weakness:

Page: 1-9

- Further clarification of the target population is needed. In some narrative, the applicant identifies people with disabilities who have diabetes as the identified population. In other cases, people with diabetes and mobility impairments is identified as the target population. In addition, the applicant identifies people with diabetes who have new onset or secondary disabilities; it is unclear why this population is identified. - An appropriate review of the literature related to the specific target population is needed.

Criterion 2: Quality of Project Design

Strength:

Page: 28-29

- Project work plan with robust anticipated outcomes is feasible and aligns with the project description, p. 28-29.

Page: 9-29, 34-38

- Based on the applicant's thorough literature review, educational and disease management interventions for the target population include in-person engagement. Contributing factors with in-person requirement will increase rates of non-compliance and unavailability, thus further substantiating the need for this type of product development. The applicant intends to use health coaching, deemed as effective, as opposed to standard approaches of texting, gaming, etc. which should lead to decreases in participation barriers with this product development, p. 9-10. Literature also supports the use of medical nutritional therapy for individuals with diabetes being proposed for this project, p. 10-11. - A six-week pilot study suggests that the individualized approach the applicant intends to use is beneficial for addressing contributing factors to diabetes, p. 14. - The technology aim, which will address accessibility and include useability testing, includes a multi-layered design to address the behavior and support challenges, p. 16-20, identified in competitive product reviews, p. 15-16. - Pilot testing using a control and two user groups, p. 20, will allow the research team and partners to measurement the fidelity and efficacy of the coaching support, p. 21-26. The sample size of 90 seems appropriate for the pilot study, p. 27-28. - Applicant's commercialization plan includes identification of future venture capital funds, p. 37, and reimbursement from Medicare and Medicaid, p. 38.

Weakness:

Page: 9-29

- The pilot study conducted by this proposal's research team was limited with a small sample size (n=13) and focused on just one community clinic in Dallas, p. 14. - While the inclusion of people with disabilities and key stakeholders is discussed, and in partnership with Lakeshore, additional detail about the demographics of the individuals and stakeholders would be helpful, p. 28. - There is limited discussion about the input from individuals with disabilities and other stakeholders into the project design, p. 9-29. - The applicant does not include one of the standard interventions for diabetes, blood glucose monitoring, in the project design, p. 9-29.

Criterion 3: Project Staff

Strength:

Page: 29-33, Attachments

- Project staff include expertise from multiple areas of diabetes interventions and behavior change management, p. 29, and project staff include members from underrepresented groups. The applicant commits to employing individuals with disabilities, p. 29-30. - The applicant intends to hire a software developer which will be key for project completion, p. 32.

Weakness:

Page: 29-33, Attachments

- The applicant does not provide information about the position of software designer given that it will be a key staff position for product development and testing, p. Attachments, p. 29-33.

Criterion 4: Adequacy and Reasonableness of the Budget

Strength:

Page: 33-34, Attachments

- Budget seems reasonable and appropriate for the project description, with appropriate allocations for project partners, p. 33-34, attachment. - Budget justification provides sufficient substantiation for line items costs, attachment.

Weakness:

Page: 33-34, Attachments

- A budget from the University of Alabama, subcontractor, would be helpful to assess the justification of this expense, Attachments.

Criterion 5: Adequacy and Accessibility of Resources

Strength:

Page: 34, 1-40, Attachments

- Applicant is a woman and minority-owned organization focused on serving underserved populations, Abstract. - Proposal includes strong partnerships with the University of Alabama, Birmingham and Lakeshore Foundation for comprehensive project expertise and experience described throughout and with appropriate budget allocations described p. 34. - Sufficient resources for project completion are described, p. 38-40.

Weakness:

None
